# Bournewood in Belfast: who's ready?

**DOI:** 10.1192/bjo.2021.640

**Published:** 2021-06-18

**Authors:** Hayley Bowes, Joseph Kane, Gillian McPherson

**Affiliations:** Belfast Health and Social Care Trust

## Abstract

**Aims:**

We aimed to determine doctors’ confidence in completing capacity assessments and reports associated with new Deprivation of Liberty Safeguards (DoLS), and the impact that these would have on practice. We hypothesised that psychiatrists would have greater confidence in completing the requisite assessments and would anticipate a greater impact on practice than doctors in other specialties.

**Background:**

On the 2ndDecember 2019 DoLS became the first part of the Mental Capacity Act (Northern Ireland) 2016 to be implemented, believed to apply to 7500 individuals across Northern Ireland (NI). As arguably the most significant change in mental health legislation in NI since 1986, the Department of Health commissioned training for all clinicians.

**Method:**

We conducted a cross-sectional survey among doctors working within psychiatry, general medicine, anaesthetics and surgery in Belfast Health and Social Care Trust prior to implementation. The survey comprised seven questions with a 10-point Likert scale. Statistical analysis included Pearson'sχ2and Spearman's rank tests.

**Result:**

79 doctors in psychiatry and 25 in other medical specialties completed the survey.Respondents were moderately confident in completing capacity assessments (median 6 (3-9)) and medical reports (median 5 (1-9)). Those that had completed training (n = 86; 83%) were significantly more confident in capacity assessment (median 7 (2-10) vs 4 (1-7); χ2(18) = 36.8, p <0.01) and medical report completion (median 5 (1-9) vs 1 (1-5); χ2(16) = 27.2, p =.04) than those that had not (n = 18; 17%). Psychiatrists had greater confidence in conducting capacity assessments (median 7(2-10)) than other doctors (median 5(1-9); χ2(9) = 18.2, p = 0.04). No significant differences were observed between the two groups with respect to medical report completion, or anticipated impact on practice.

Respondents who most frequently conducted capacity assessments as part of their current practice anticipated higher degrees of impact on their individual practice (rs = 0.51, p < 0.01) and their service (rs = 0.50, p < 0.01)

**Conclusion:**

Engagement with the commissioned training was encouraging. Respondents were, on average, relatively confident in conducting capacity assessment, but considerable variation in confidence, and a lower confidence in completing medical reports. This might suggest that some may require further training. A poor response rate among non-psychiatrists indicates potential respondent bias in favour of those already more cognisant of capacity in routine practice. A correlation between more practiced assessors and anticipated impact on service provision could suggest that some clinicians may be underestimating the potential impact of DoLS; the same groups should therefore be resurveyed after DoLS implementation.

